# A206 COMBINED EFFECT OF TOBACCO SMOKING AND ORAL CONTRACEPTIVE USE ON THE RISK OF INFLAMMATORY BOWEL DISEASE

**DOI:** 10.1093/jcag/gwae059.206

**Published:** 2025-02-10

**Authors:** C Fantodji, P Jantchou, M Rousseau

**Affiliations:** Institut national de la recherche scientifique, Quebec, QC, Canada; Centre Hospitalier Universitaire Sainte-Justine, Montreal, QC, Canada; Institut national de la recherche scientifique, Quebec, QC, Canada

## Abstract

**Background:**

Tobacco and oral contraceptives are involved in the development of inflammatory bowel disease through their effect on the intestinal immune system. Oral contraceptives may modify the effect of tobacco by increasing nicotine absorption.

**Aims:**

We investigated the independent and combined effects of smoking and oral contraceptives use on the occurrence of Crohn’s disease (CD) and ulcerative colitis (UC).

**Methods:**

This case-control study, nested within the Quebec Birth Cohort on Immunity and Health (CO-MMUNITY), included persons born in Quebec in 1970-1974 and followed until 2014. Cases of CD and UC were identified using validated algorithms based on administrative health data. All cases and randomly selected controls were invited to participate. Smoking and oral contraceptive use were documented using self-reported questionnaires. We used weighted incidence density sampling to treat time-dependent variables. We performed weighted Cox proportional hazards models to estimate adjusted hazard ratios (HR) and 95% confidence intervals (95% CI). All multiple regression models were adjusted for relevant covariates identified using directed acyclic graphs. Additive and multiplicative interactions between smoking and oral contraceptive use were assessed.

**Results:**

Among the participating women, there were 590 controls, 790 cases of CD and 336 cases of UC. The median (interquartile range) age at IBD diagnosis was years 24.7 (20.7-30.5) for CD cases and 26.2 years (21.5-31.9) for UC cases. Forty-three percent of controls were ever smokers compared to 59% of CD cases and 45% of UC cases. Most participants (~91% per strata) used oral contraceptives. Smoking increased the risk of CD (adjusted HR=1.68; 95% CI: 1.35-2.09) and decreased the risk of UC (adjusted HR=0.56; 95% CI: 0.40-0.77). Oral contraceptive use increased the risk of CD by 33% (adjusted HR=1.33; 95% CI: 1.10-1.61) but not UC (adjusted HR=1.09; 95% CI: 0.84-1.41). There was an increased risk of CD in smokers who used oral contraceptives compared to non-smokers and non-users of oral contraceptives (adjusted HR=2.23; 95% CI: 1.66-2.98). The association between smoking and CD did not differ by oral contraceptive use (*p* for multiplicative interaction: 0.9; *p* for additive interaction: 0.6) or ulcerative colitis (*p* for multiplicative interaction: 0.1; *p* for additive interaction: 0.4).

**Conclusions:**

Our results suggest independent effects of smoking and oral contraceptive use on the occurrence of CD and UC. Although the risk of CD was higher in women who smoked and used oral contraceptives, no additive or multiplicative interactions were found for either CD or UC.

**Funding Agencies:**

CIHRCanada Foundation for Innovation & the Québec Ministry of Education, Leisure and Sports (#12532), Fonds de recherche du Québec-Santé (FRQS, #16227), Multiple Sclerosis Society of Canada (#2435), Institut de la statistique du Québec, Fonds de recherche du Québec-Santé (#326758), Fonds de recherche du Québec-Nature Technologie (#334829), Regroupement intersectoriel de recherche en santé de l’Université du Québec, Quebec inter-University Centre for Social Statistics, Fondation de l’INRS

